# Sex and Gender in COVID-19 Vaccine Research: Substantial Evidence Gaps Remain

**DOI:** 10.3389/fgwh.2021.761511

**Published:** 2021-11-01

**Authors:** Amy Vassallo, Sultana Shajahan, Katie Harris, Laura Hallam, Carinna Hockham, Kate Womersley, Mark Woodward, Meru Sheel

**Affiliations:** ^1^The George Institute for Global Health, University of New South Wales, Sydney, NSW, Australia; ^2^The George Institute for Global Health, School of Public Health, Imperial College London, London, United Kingdom; ^3^University of Edinburgh, NHS Lothian, Edinburgh, United Kingdom; ^4^National Centre for Epidemiology and Population Health, Research School of Population Health, ANU College of Health and Medicine, Australian National University, Canberra, ACT, Australia

**Keywords:** COVID-19, vaccine, immunisation, evidence, gender equality, safety, gender

## Abstract

Since the start of the COVID-19 pandemic there has been a global call for sex/gender-disaggregated data to be made available, which has uncovered important findings about COVID-19 testing, incidence, severity, hospitalisations, and deaths. This mini review scopes the evidence base for efficacy, effectiveness, and safety of COVID-19 vaccines from both experimental and observational research, and asks whether (1) women and men were equally recruited and represented in vaccine research, (2) the outcomes of studies were presented or analysed by sex and/or gender, and (3) there is evidence of sex and/or gender differences in outcomes. Following a PubMed search, 41 articles were eligible for inclusion, including seven randomised controlled trials (RCTs), 11 cohort studies, eight cross-sectional surveys, eight routine surveillance studies, and seven case series. Overall, the RCTs contained equal representation of women and men; however, the observational studies contained a higher percentage of women. Of 10 studies with efficacy data, only three (30%) presented sex/gender-disaggregated results. Safety data was included in 35 studies and only 12 (34%) of these presented data by sex/gender. For those that did present disaggregated data, overall, the majority of participants reporting adverse events were women. There is a paucity of reporting and analysis of COVID-19 vaccine data by sex/gender. Research should be designed in a gender-sensitive way to present and, where possible analyse, data by sex/gender to ensure that there is a robust and specific evidence base of efficacy and safety data to assist in building public confidence and promote high vaccine coverage.

## Introduction

The important influence of sex and gender on health has come to the forefront during the COVID-19 pandemic. Globally, shared sex-disaggregated data has led to important understanding about COVID-19 testing, incidence, severity, hospitalisations, and deaths (1–4). For example, while the proportion of COVID cases in women and men are roughly equal, men have around three times the odds of intensive care admission and a 40% higher odds of dying from COVID-19 than women (4). Known biological differences in adaptive and innate immune responses between sexes explain some of these observed differences (4). Socio-cultural gender constructs also influence these outcomes through differing exposures to the disease (such as high occupational exposure in frontline healthcare workers, who are predominantly women), risk factors for severe disease (such as higher smoking rates in men), existence of comorbidities, and engagement with healthcare services for prevention, detection, and treatment (typically lower in men) (5–8).

Sex and gender are also important factors in understanding immunisation, including vaccine delivery, efficacy, and frequency and severity of adverse reactions (6). Sex and gender differences in immunisation outcomes have been observed across age groups for other vaccine preventable diseases, with women typically developing higher antibody responses, and reporting more local and systemic adverse reactions, compared with men (9). These differences have been observed in response to vaccines using different technologies, including the Calmette-Guerin vaccine, measles, mumps and rubella, yellow fever, and influenza vaccines (10). Several biological mechanisms have been proposed, including immunological, hormonal, genetic, and microbiota differences between females and males (10, 11).

Developing an effective vaccine against COVID-19 has been a global research priority, with several different vaccines administered on a large scale across the globe in 2020 and 2021 as part of national immunisation programs. This shift from experimental to observational (including routine surveillance) research provides valuable acceptability, effectiveness, and real world safety data (12). Effective, efficient, equitable, and publicly acceptable immunisation programs are needed for control of the COVID-19 pandemic globally. These programs need to be rooted in sex- and gender-sensitive evidence. The aim of this mini review is to scope the evidence base for efficacy, effectiveness and safety of COVID-19 vaccines, and whether (1) women and men were equally recruited and represented in each vaccine's research, (2) the outcomes of studies were presented or analysed by sex and/or gender, and (3) there is evidence of sex and/or gender differences in outcomes.

## Methods

### Search Strategy and Selection Criteria

We searched PubMed for peer-reviewed literature on the efficacy, effectiveness, and/or safety of COVID-19 vaccines included in the COVID-19 Vaccines Global Access (COVAX) portfolio as of 6 May 2021: Pfizer/BioNTech (BNT162b2), Oxford/AstraZeneca (AZD1222), Novavax (NVX-CoV2373), Covovax (NVX-CoV2373), Johnson&Johnson (Ad26.COV2.S), Sanofi/GSK (VAT00002), and Moderna (mRNA-1273) (13, 14). Search terms relating to vaccine name, SARS-CoV-2, COVID-19, COVID-19 vaccines, phase 3 and 4 clinical trials, efficacy, effectiveness and mass vaccination, and relevant synonyms of these, were used (see Appendix 1 for detailed search strategy). To identify articles containing safety data, additional search terms relating to adverse effects, safety monitoring, safety profile, and appropriate synonyms, were used. Searches were conducted using standard keywords as well as MeSH terms.

Eligible studies included phase 2/3 or 3 randomised controlled trials (RCTs) (experimental studies) and post-market observational studies including cohort studies, cross-sectional studies, routine surveillance reports, and case series. Animal studies, phase 1 and/or 2 RCTs and case studies were excluded. All article types that presented original data were included, including research articles, editorials, responses, and letters to the editor. Immunogenicity data were excluded, however any relevant safety outcome data from these studies were included. In case of duplicate publications containing the same data, the report with the greatest amount of data or the one published first was included.

### Data Extraction and Analysis

Title/abstract screening, full-text review and data extraction were conducted in duplicate by AV (all papers) and SS, KH, LH, and CH. Any disagreements between reviewers were resolved by discussion. Data were extracted for study title, author, date of publication, vaccine name(s), study design, population subgroup (e.g. healthcare workers, people with pre-existing conditions), percentage of women and men participants, vaccine efficacy and effectiveness (as defined by study authors), and the percentage of women and men who presented with adverse events (as defined by study authors). Results data for up to 12 adverse events per article were extracted. Care was taken not to report the occurrence of an adverse event as “zero” unless it was explicitly stated as such in the article (15). We were unable to distinguish between sex and gender based on the included studies, so hereafter refer to sex/gender (16, 17).

For all studies, we examined the reported sex/gender distribution of the research participants. The number of participants in a study was derived from the number of participants reported at the study end point (specifically those on whom the study was conducted); if this was not available the number at study baseline was used. If data were presented by vaccine dose in the same participant group, then the number of participants in the second dose was extracted. Participant number from national surveillance data was taken as the number of vaccine recipients within the reporting period of the study (i.e., CDC reports: 14–23 December 2020 for Pfizer, and 21 December 2020 to 10 January 2021 for Moderna).

For all studies, we examined whether efficacy or effectiveness (hereafter efficacy) and safety data were presented by sex/gender. Studies were marked “Yes” for sex/gender-disaggregated data if they presented disaggregated data for all their reported main outcomes, either in the main results or Supplementary Information. For studies other than case series that disaggregated their safety findings by sex/gender, we either extracted relative risks of adverse events in women and men, or calculated them where possible from presented sex/gender-disaggregated participant and outcome data, in order to summarise the evidence for significant sex/gender differences.

No meta-analyses were performed owing to the relatively small number of studies available, and the heterogeneity in efficacy and safety outcomes that were reported.

## Results

A total of 323 relevant studies were identified, and 41 were eligible for inclusion in this review (Appendix Figure 1). Included studies presented data on the following vaccines: Oxford/AstraZeneca (*n* = 11), Pfizer/BioNTech (*n* = 28), Moderna (*n* = 12), Johnson&Johnson (*n* = 2), with some studies reporting data for more than one vaccine type.

Table 1 presents a summary of the content, participants, and presentation of outcomes in each of the included studies. Two adjusted for sex in their vaccine effectiveness model (30, 51), one study included sex-matched controls (43) and several articles were published as research letters and correspondence, rather than full research articles.

**Table 1:** Description of studies and their inclusion of sex/gender-disaggregated data.

				**Efficacy/Effectiveness data**		**Safety data**		
**Study Author (year) Journal**	**Population subgroup (if any)**	**Total n per study**	**%. of women participants**	**Contains efficacy/** **effectiveness data**	**Sex/gender-** **disaggregated outcomes**	**Contains** ** safety data**	**Sex/gender-disaggregated outcomes**	**List of adverse** ** reaction outcomes** ** investigated**
**Randomised controlled trials (*****n*** **= 7)**		**88,255**	**50**	**6**	**2**	**5**	**0**	
Baden et al. (18) N Engl J Med	–	30,351	47	Yes	Yes	Yes	No	Local grade 1, 2, or 3 adverse reactions: Any, pain, erythema, swelling, axillary swelling/tenderness. Systemic grade 1, 2, or 3 adverse reactions: Any, fever, headache, fatigue, myalgia, arthralgia, nausea/vomiting, chills.
Emary et al. (19) Lancet	–	8,534	59	Yes	No	No	NA	NA
Frenck et al. (20) N Engl J Med	–	2,260	49	Yes	No	Yes	No	Local mild, moderate, severe, and grade 4 adverse reactions: Pain at injection site, redness, swelling. Systemic mild, moderate, severe and grade 4 adverse reactions: Fever, fatigue, headache, chills, muscle pain, vomiting, diarrhoea, joint pain.
Madhi et al. (21) N Engl J Med	–	2,021	43	Yes	No	Yes	No	General disorders, administration site conditions, infections, nervous system, respiratory, gastrointestinal, musculoskeletal, skin, reproductive system, eye, vascular, metabolism, ear, immune system, renal, blood, psychiatric disorders, and severe adverse events.
Polack et al. (22) N Engl J Med	–	37,706	49	Yes	Yes	Yes	No	Local mild, moderate, severe and grade 4 adverse reactions: Pain at injection site, redness, swelling. Systemic mild, moderate, severe and grade 4 adverse reactions: Fever, fatigue, headache, chills, vomiting, diarrhoea, muscle pain, joint pain.
Ramasamy et al. (23) Lancet	–	552	51	No	NA	Yes	No	Local mild, moderate and severe adverse reactions: Induration, itch, pain, redness, swelling, tenderness, warmth. Systemic mild, moderate and severe adverse reactions: Chills, fatigue, fever, headache, joint pain, malaise, muscle ache, nausea.
Voysey et al. (24) Lancet	–	6,831	62	Yes	No	No	NA	NA
**Cohort study (*****n*** **= 11)**		**1,555,243**	**56**	**2**	**1**	**9**	**4**	
Achiron et al. (25) Mult Scler	People with multiple sclerosis	435	65	No	NA	Yes	No	Pain at injection site, fever/chills/flu-like symptoms, fatigue, headache, muscle or joint pain, new or worsening neurological symptomatology, face tingling, acute MS relapse.
Bae et al. (26) J Korean Med Sci	Healthcare workers	5,866	76	No	NA	Yes	Yes	Local pain, redness, swelling, fever, fatigue, headache, chills, vomiting, diarrhoea, muscle ache, joint pain.
Bernstine et al. (27) Clin Nucl Med	People with cancer	256	54	No	NA	Yes	Yes	Hypermetabolic axillary lymph nodes
Blumenthal et al. (28) JAMA	Hospital workers	64,900	NR	No	NA	Yes	Yes	Anaphylaxis, acute allergic reactions
Dagan et al. (29) N Engl J Med	Health service employees	1,193,236	50	Yes	Yes	No	NA	NA
Fabiani et al. (30) Euro Surveill	Healthcare workers	6,423	78	Yes	No	No	NA	NA
Jeon et al. (31) J Korean Med Sci	Healthcare workers	994	77	No	NA	Yes	Some^+^	Fatigue, headache, malaise, arthralgia, chills, fever, nausea/vomiting, diarrhoea, local tenderness, redness, swelling, resting pain.
Kim et al. (32) J Korean Med Sci	Healthcare workers	1,511	72	No	NA	Yes	Some^*^^+^	Pain at injection site, redness/swelling at injection site, lymphadenopathy, fever, chills, fatigue, nausea, vomiting, headache, myalgia, arthralgia, urticaria.
Krammer et al. (33) N Engl J Med	–	230	68	No	NA	Yes	No	Pain at injection site, swelling at injection site, erythema, fatigue, headache, chills, muscle pain, fever, joint pain.
Pimpinelli et al. (34) J Hematol Oncol	People with hematologic malignancies	128	48	No	NA	Yes	No	Pain, tenderness, fever, headache, malaise, myalgia, chills.
Pottegard et al. (35) BMJ	–	281,264	79	No	NA	Yes	Yes	Arterial events, venous thromboembolism,/coagulation disorders, bleeding events.
**Cross-sectional survey (*****n*** **= 8)**		**7,243**	**77**			**8**	**0**	
Boyarsky et al. (36) Transplantation	Solid organ transplant recipients	187	69	No	NA	Yes	No	Pain, redness, swelling, fever, chills, fatigue, headache, myalgia.
El-Shitany et al. (37) Int J Gen Med	–	455	64	No	NA	Yes	Some^+^	Arm pain, injection site pain, injection site swelling and redness, fever, chills, fatigue, headache, nausea and vomiting, diarrhoea, muscle pain, joint pain.
Kadali et al. (38) Int J Infect Dis	Healthcare workers	803	87	No	NA	Yes	No	Generalised, weakness/fatigue, headache, chills, localised swelling at injection site, muscle pain/myalgia, arthritis/joint pain, diarrhoea, vomiting, fever, nausea, sore arm/pain, sweating.
Nittner-Marzalska et al. (39) Vaccines	Medical professionals and medical students	1,707	79	No	NA	Yes	No	Fever, arthralgia, myalgia, headache, palpitations, vomiting, local swelling, local redness, local pain, allergic reactions.
Riad et al. (40) J Clin Med	Healthcare workers	877	88	No	NA	Yes	No	General side effects: injection site pain, fatigue, headache, muscle pain, chills, joint pain, injection site swelling, injection site redness feeling unwell, lymphadenopathy, nausea.Oral side effects
Song et al. (41) J Korean Med Sci	Healthcare workers	2,478	76	No	NA	Yes	No	Injection site pain, injection site erythema, fever, headache, myalgia, arthralgia, fatigue, nausea/vomiting, rash, limitation of arm movement, facial paraesthesia, chill.Anaphylactoid reaction.
Sørvoll et al. (42) J Thromb Haemost	Healthcare workers	602	71	No	NA	Yes	No	Thrombocytopenia, anti-PF4/PVS reaction antibodies. Fever, headache, vomiting, fatigue, cutaneous bleeding, malaise, muscle/joint ache.
Waissengren et al. (43) Lancet Oncol	People with cancer	134	46	No	NA	Yes	No	Pain at injection sites, fatigue, headache, muscle pain, chills, fever, gastrointestinal complications, flu-like symptoms, local rash, local swelling.
**Routine surveillance (*****n*** **= 8)**		**41,104,426**	**61**	**2**	**0**	**6**	**3**	
CDC COVID-19 Response Team et al. (44) Morb Mortal Wkly Rep	VAERS	1,893,360	62	No	NA	Yes	Yes	Anaphylaxis, non-anaphylaxis allergic reactions
CDC COVID-19 Response Team et al. (45) Morb Mortal Wkly Rep	VAERS	4,041,396	61	No	NA	Yes	Yes	Anaphylaxis, non-anaphylaxis allergic reactions
Gee et al. (46) Morb Mortal Wkly Rep	VAERS and V-safe	1,629,065	NR	No	NA	Yes	Some^+^	Pain at injection site, fatigue, headache, myalgia, chills, fever, swelling at injection site, joint pain, nausea.
Hause et al. (47) Morb Mortal Wkly Rep	VAERS	7,988,624	NR	No	NA	Yes	Some^+^	Anxiety related adverse events: chest pain, light-headedness or dizziness, nausea/vomiting, pallor or diaphoresis, syncope, tachycardia, seizure-like activity, hypotension.
Shay et al. (48) Morb Mortal Wkly Rep	VAERS and V-safe	7,980,000	NR	No	NA	Yes	Some^*^	Non-serious, serious events, injection site reactions, systemic reactions, health impacts, fatigue, injection site pain, headache, myalgia, fever, joint pain, nausea, diarrhoea.
Shimabukuro et al. (49) JAMA	VAERS	17,524,676	NR	No	NA	Yes	Yes	Anaphylaxis
Skowronski et al. (50) N Engl J Med	Documents submitted to Food and Drug Administration	43,355	NR	Yes	No	No	NA	NA
Thompson et al. (51) Morb Mortal Wkly Rep	Healthcare workers in HEROES-RECOVER	3,950	62	Yes	No	No	NA	NA
**Case Series (*****n*** **= 7)**		**154**	**71**			**7**	**5**	
Farinazzo et al. (52) J Eur Acad Dermatol Venereol	–	46	89	No	NA	Yes	Yes	Cutaneous adverse reaction, any adverse event
Fernandez-Prada et al. (53) Euro Surveill	Healthcare workers	20	100	No	NA	Yes	Yes	Supraclavicular lymphadenopathy
Johnston et al. (54) JAMA Dermatol	–	16	81	No	NA	Yes	Yes	Localised cutaneous reaction (injection site reactions)
Lee et al. (55) Am J Hematol	–	20	40	No	NA	Yes	Some^*^	Thrombocytopenia, bruising, bleeding
Meylan et al. (56) Hypertension	–	9	78	No	NA	Yes	Yes	Stage 3 hypertension
Roman et al. (57) Front Immunol	–	43	47	No	NA	Yes	Some^*^	Quadriplegia, paraplegia, acute disseminated encephalomyelitis, spinal cord lesions
Shemer et al. (58) Isr Med Assoc J	–	9	NR	No	NA	Yes	Yes	Acute-onset facial nerve palsy

*NR, Not reported; NA, not applicable*.

* *Study sex/gender-disaggregated presentation of some but not all safety outcomes*.

+ *Study presented sex/gender-disaggregated summary results*.

Across all seven RCTs, there was a 50/50 distribution in the sex/gender of participants (Table 1): one study included 44% women, four studies included 45–55% women, and two studies ~60% women. A total of 3/11 cohort studies included 45–55% women, with the other seven including more women (65–79%), and one not reporting. In the case of cross-sectional studies, one study included 46% women and the remainder included 64–88% women. For routine surveillance reports, 5/8 (63%) did not provide any sex/gender-disaggregated percentages of participants, and the remaining three included 61–62% women. Of the seven case studies, one did not provide disaggregated participant data, one included 100% women, and the rest included 40–89% women.

Of the 10 studies investigating vaccine efficacy, three (two RCTs and one cohort) included sex/gender-disaggregated results (Table 1). None of these studies reported a significant difference in their primary efficacy outcome between women and men.

**Figure 1:**
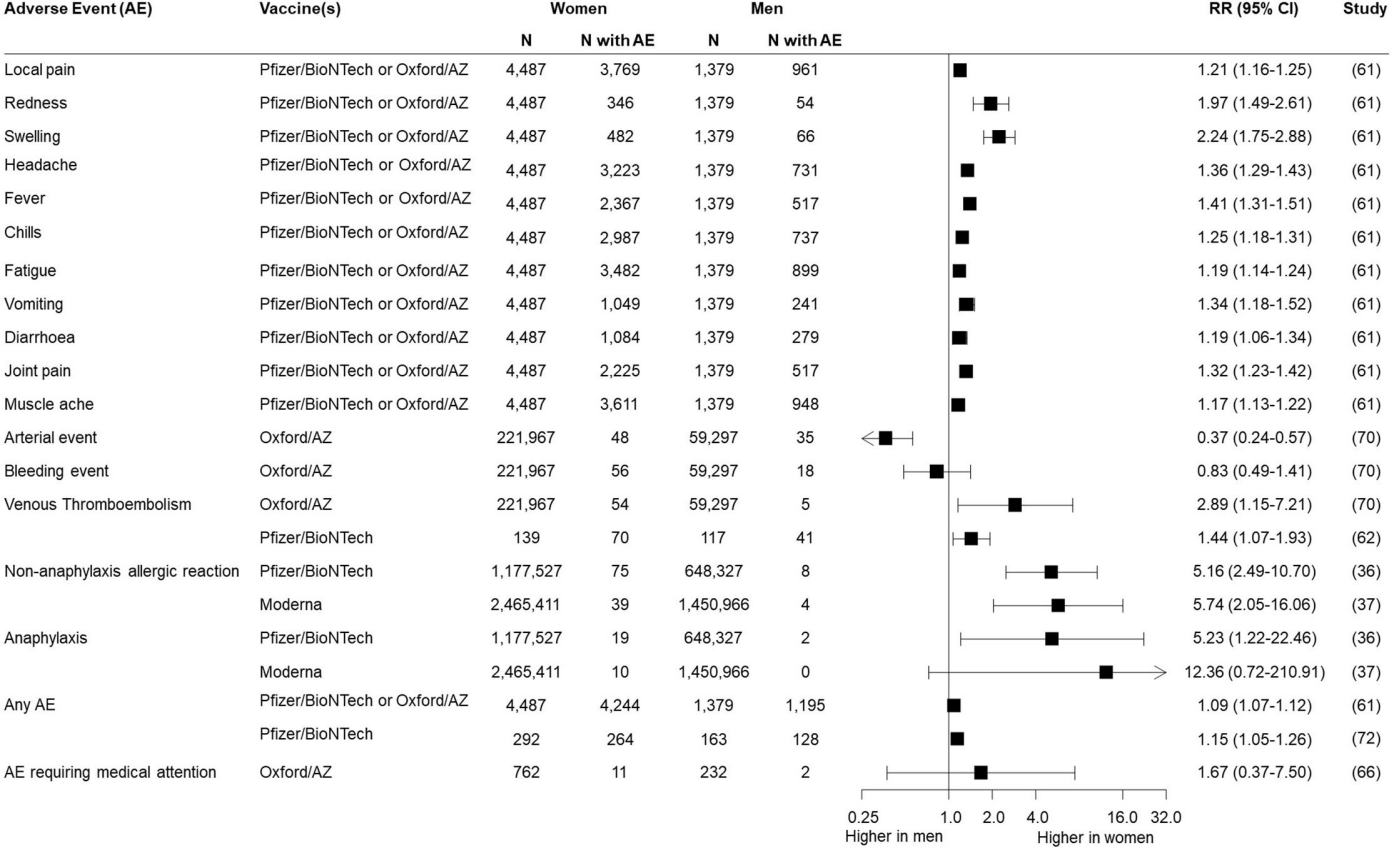
Risk of adverse events following COVID-19 immunisation reported in women and men.

A range of local and systemic adverse reactions following immunisation were reported, as indicated in Table 1. Of the 35 experimental and observational studies containing safety data, 12 (34%) disaggregated all their outcomes by sex/gender, none of which were RCTs and five of which were case series. An additional four studies reported sex/gender-disaggregated data for some but not all of their outcomes, and five additional studies presented summary statements by sex/gender, such as overall percentages of women and men experiencing at least one adverse event, or percentage requiring emergency department presentation. Sex/gender-specific risks of different adverse events were available from seven studies (Figure 1), which often indicated a higher risk reported for women. This included typical local and systemic reactions such as redness [RR = 1.97 (95% CI: 1.49–2.61)], swelling [RR = 2.24 (95% CI: 1.75–2.88)], and fever [RR = 1.41 (95% CI: 1.31–1.51)] as well as non-anaphylaxis allergic reaction [RR = 5.16 (95% CI: 2.49–10.70) for Pfizer/BioNTech vaccine and RR = 5.74 (95% CI: 2.05–16.06) for Moderna vaccine]. Risk of one reaction, arterial event, appeared lower in women [RR = 0.37 (95% CI: 0.24–0.57)].

No study justified lack of sex/gender-disaggregated data. However, two studies did acknowledge the dominance of women in their samples of healthcare workers (23, 30, 40, 43, 51).

## Discussion

### Overall Summary of Findings

In this mini review of studies reporting efficacy and/or safety outcomes of vaccines included under COVAX, we found that women and men were equally represented in RCTs, whilst women (and healthcare workers) comprised the majority of participants in observational studies. Despite global calls for the routine disaggregation of COVID-19 data by sex/gender (59–62), only two RCTs reported efficacy data by sex/gender, and none stratified safety data by sex/gender. Among the 34 included observational studies, 13 (38%) presented all sex/gender-disaggregated data (*n* =1 (3%) for efficacy and *n* = 12 (35%) for safety). There was no evidence of sex/gender differences in vaccine efficacy, yet a higher risk of adverse events were reported among women compared to men. However, this evidence was limited in terms of both number and size of studies, which may not have been specifically designed to detect a difference between groups.

Our review findings demonstrate a disappointing, and potentially detrimental, lack of sex/gender-specific evidence across study types of the COVID-19 vaccine experimental research studies as well as observational reporting.

### Representation by Sex/Gender in Research

We found equal representation of women and men in COVID-19 vaccine RCTs. This is despite another COVID-19 review finding that less than half of registered vaccine trials explicitly mentioned sex/gender in their recruitment strategy as part of their ClinicalTrials.gov registration (63). Therefore, our finding may be due to specific efforts by the research team to ensure equal recruitment, or because some of the usual barriers to women's participation in research, such as belief in the relevance of the health problem, concerns about risk, and trial logistical burden, may not have been as pervasive (7, 64). Women made up the majority of participants in non-RCT studies in our review. This is likely due, in part, to risk-based prioritisation of vaccine rollouts, which meant that healthcare and hospital workers, primarily women, were amongst the first to be vaccinated. For case series, it might reflect gender differences in reporting.

### Reporting of Sex/Gender-Disaggregated Data

Our study identified a lack of sex/gender-disaggregated reporting or sub-group analyses in COVID-19 vaccine research. Despite roughly equal representation in RCTs, only a third of studies reported sex/gender-disaggregated efficacy data, and none reported safety data. This lack of focus on sex/gender aligns with findings of a recently published review of COVID-19 clinical trials of drug-based and biological/vaccine interventions, which found that only 18% of trials reported sex-disaggregated results or subgroup analyses (63). Another review published in early 2021 concluded that there was inadequate reporting of sex/ gender in COVID-19 clinical studies, that main outcomes were rarely reported or analysed by sex/gender, and this absence was seldom justified (65). We recognise the challenges in ensuring adequate sample sizes in clinical research to conduct subgroup analyses, particularly when considering rarer adverse events. However, the presentation of sex/gender-disaggregated data, either in main results or Supplementary Appendices as recommended in the SAGER guidelines (66), will be an asset for facilitating future meta-analyses as the pandemic progresses and the volume of COVID-19 vaccine research increases.

A novel element of our review is the inclusion of multiple study designs, not only RCTs. The complete absence of sex/gender-disaggregated safety data in COVID-19 vaccine trials means that post-marketing surveillance of sex/gender-specific adverse events is particularly important. Yet there was still an absence of reporting, with sex/gender-disaggregated adverse event data available in only four cohort and three national surveillance studies. While age and sex/gender data are typically collected through routine national surveillance systems (67), and shared with decision makers, the lack of data in the public domain has consequences for immunisation program delivery and uptake – further discussed below.

### Evidence of Sex/Gender Differences

Of the seven studies where sex/gender-specific risk was reported, a higher risk of certain types of adverse events were observed among women. A large prospective observational study published subsequently to our searches found that local and systemic side effects were self-reported at lower frequencies than reported in RCTs, and minor events such as headache and fatigue were more common in women (68). These findings align with that of other vaccines (69), and are likely primarily due to women being more likely to report their symptoms than men (70, 71). Looking at anaphylactic and non-anaphylactic allergic reactions, although rare, 90% were reported in women (44, 45, 49). This is likely influenced by greater percentage of women being vaccinated (45). These findings raise important questions around the gendered dimensions of immunisation, and demonstrate the value of routine collection and analysis of sex/gender-disaggregated data for further investigation of trends and mitigation strategies.

### Consequences of a Lack of Sex/Gender Specific Evidence

Lack of sex/gender data in immunisation, including efficacy, safety and coverage data, has been a longstanding issue, not only one of the COVID-19 pandemic. Yet sex/gender has a critical influence on immunisation outcomes, at individual, household, community, health system, and policy levels (72). The lack of incorporation of sex/gender in COVID-19 vaccine research, as demonstrated across the spectrum of research designs in this review, results in an evidence base that does not lend itself to effective public communication around the utility and safety of vaccines. One current example is vaccine hesitancy and the slow uptake in some regions (73, 74). While a complex issue with many inter-related factors, concerns around safety (including pain) and misconceptions about effectiveness and side effects are known to be critical influences of vaccine hesitancy and delay (6, 75). Previous research has fairly consistently found that men report a higher intention to vaccinate than women (76–78), though intention does not always reflect action or access. Therefore, an increase in sex/gender-specific information in the public domain would appear to be a prudent approach so as to help address misconceptions and mitigate vaccine hesitancy. This tailored advice is only possible if research pays more attention to sex/gender, and the particular concerns of women, men, and non-binary people.

A gendered lens should also be applied when designing research, including determining what data to collect. This includes consideration of how research design and conduct may be explicitly or implicitly sex/gender-biased, for example through exclusion of those who are pregnant or breastfeeding (79), or how research may potentially exacerbate existing sex/gender-related disparities or knowledge gaps. As an example, only one of the included studies examined adverse events related to the reproductive system, and the authors did not disaggregate these findings by sex/gender, or by age (21). Surveillance studies have also not reported data on menstrual irregularities or fertility. This may limit the ability of scientists and doctors to effectively respond to anecdotal reports and concerns within the community about such side effects post-immunisation, which have been amplified online and by the media (80–83). Greater attention to sex/gender in the design of COVID-19 vaccine research may help to address this data gap, allowing for improved public communication about adverse events with patients—particularly women.

## Recommendations for Future Immunisation Research and Surveillance

There are some limitations to this mini-review. In particular, the potential for missing studies, as only one database was searched and COVID-19 publications are rapidly increasing in number over time. However, this snapshot demonstrates an important evidence gap and discusses how adopting a sex/gender lens to data collection, reporting and analysis can have benefits for vaccination program outcomes. This mini-review focused only on sex/gender, without incorporating other intersectional factors, such as age and ethnicity, that may have an important impact on COVID-19 immunisation and outcomes and should be incorporated into research. Further, a quality assessment of articles was not conducted, which could be relevant for studies reporting sex/gender differences in outcomes, particularly regarding sample size and gender distribution. However, this mini review highlights substantial gaps in sex/gender-specific COVID-19 vaccine research.

Sex/gender, and other intersecting factors, impact how people are experiencing the COVID-19 pandemic (84). Therefore, research, policy and recommendations for COVID-19 vaccination must consider sex/gender in order to achieve optimally effective and equitable outcomes. Based on our findings, we present the following recommendations for future research:

- All research studies should, within reason, include a sex/gender lens in their research design and recruitment, sex/gender-disaggregate their main outcomes and, where feasible, analyse potential sex/gender-based differences, or indeed similarities. This aligns with advice provided to the WHO on critical considerations for equitable COVID-19 vaccine research, development, and delivery (6).
- Data from research studies that collect the sex/gender of participants, but are not statistically powered to analyse results by sex/gender, should nevertheless make sex/gender results publicly available for pooling in evidence syntheses, even if only through Supplementary Data published online (66, 85).
- Medical journals and editors, as well as public health bodies, should redouble their efforts in enforcing recognition of sex/gender in reporting of COVID-19 research (65, 86) including enforcement of policies or endorsed guidelines and instructions and advice for peer reviewers
- Public health data systems, processes, and platforms should be established or adapted to collect, publicly report, and reflect on sex/gender-disaggregated outcomes from nationwide/mass immunisation programs (87).

## Conclusion

Studies developing new vaccines or investigating their impact in populations should be designed and implemented in a sex/gender-sensitive way. Failure to recognise important sex/gender implications on efficacy, safety, and implementation will be detrimental to the global vaccine rollout, and ultimately control of the COVID-19 pandemic. Review of clinical trial data highlights missed opportunities to apply a sex/gender-sensitive lens in the development of COVID-19 vaccines. Public health data gathered through routine surveillance should be sex/gender-disaggregated and made publicly available to increase reliability of data, drive public confidence in immunisation programs, decrease vaccine hesitancy, and increase coverage.

## Author Contributions

AV conceived the study and wrote the initial draft. SS designed and conducted the searches in consultation with AV. AV, SS, KH, LH, and CH conducted the data screening, extraction, and analysis. SS, KH, LH, CH, MW, KW, and MS critically reviewed the study plan and manuscript and rewrote sections. All authors contributed to manuscript revisions, read, and approved the submitted version.

## Funding

MS was funded by a Westpac Research Fellowship.

## Conflict of Interest

MW is a consultant to Amgen, Kyowa Kirin, and Freeline. The remaining authors declare that the research was conducted in the absence of any commercial or financial relationships that could be construed as a potential conflict of interest.

## Publisher's Note

All claims expressed in this article are solely those of the authors and do not necessarily represent those of their affiliated organizations, or those of the publisher, the editors and the reviewers. Any product that may be evaluated in this article, or claim that may be made by its manufacturer, is not guaranteed or endorsed by the publisher.
