# Supplementary material for: Sex and Gender in COVID-19 Vaccine Research: Substantial Evidence Gaps Remain
Source: Front Glob Womens Health. 2021 Nov 1;2:761511. doi: 10.3389/fgwh.2021.761511 (PMC8593988; doi:10.3389/fgwh.2021.761511)
Supplement: Supplementary file 1 [file Data_Sheet_1.pdf]

Supplementary Material

1 Search strategies conducted in PubMed

Vaccine efficacy/effectiveness search strategy

Pfizer/BioNTech BNT162b2

"BNT162b2"[Title/Abstract] AND ("COVID-19"[MeSH Terms] OR "COVID-19 Vaccines"[MeSH Terms] OR "SARS-CoV-2"[MeSH Terms]) AND ("clinical trials, phase iii as topic"[MeSH Terms] OR "clinical trials, phase iv as topic"[MeSH Terms] OR "Clinical Trials as Topic"[MeSH Terms] OR "phase 3"[Title/Abstract] OR "phase 4"[Title/Abstract] OR "efficacy"[Title/Abstract] OR "effectiveness"[Title/Abstract] OR "phase III"[Title/Abstract] OR "phase IV"[Title/Abstract] OR "mass vaccination"[Title/Abstract])

Oxford/AstraZeneca AZD1222

"AZD1222"[Title/Abstract] AND ("COVID-19"[MeSH Terms] OR "COVID-19 Vaccines"[MeSH Terms] OR "SARS-CoV-2"[MeSH Terms]) AND ("clinical trials, phase iii as topic"[MeSH Terms] OR "clinical trials, phase iv as topic"[MeSH Terms] OR "Clinical Trials as Topic"[MeSH Terms] OR "phase 3"[Title/Abstract] OR "phase 4"[Title/Abstract] OR "efficacy"[Title/Abstract] OR "effectiveness"[Title/Abstract] OR "phase III"[Title/Abstract] OR "phase IV"[Title/Abstract] OR "mass vaccination"[Title/Abstract])

Novavax NVX-CoV2373/ Covovax NVX-CoV2373

"NVX-CoV2373"[Title/Abstract] AND ("COVID-19"[MeSH Terms] OR "COVID-19 Vaccines"[MeSH Terms] OR "SARS-CoV-2"[MeSH Terms]) AND ("clinical trials, phase iii as topic"[MeSH Terms] OR "clinical trials, phase iv as topic"[MeSH Terms] OR "Clinical Trials as Topic"[MeSH Terms] OR "phase 3"[Title/Abstract] OR "phase 4"[Title/Abstract] OR "efficacy"[Title/Abstract] OR "effectiveness"[Title/Abstract] OR "phase III"[Title/Abstract] OR "phase IV"[Title/Abstract] OR "mass vaccination"[Title/Abstract])

J&J Ad26.COV2.S

"Ad26.COV2.S"[Title/Abstract] AND ("COVID-19"[MeSH Terms] OR "COVID-19 Vaccines"[MeSH Terms] OR "SARS-CoV-2"[MeSH Terms]) AND ("clinical trials, phase iii as topic"[MeSH Terms] OR "clinical trials, phase iv as topic"[MeSH Terms] OR "Clinical Trials as Topic"[MeSH Terms] OR "phase 3"[Title/Abstract] OR "phase 4"[Title/Abstract] OR "efficacy"[Title/Abstract] OR "effectiveness"[Title/Abstract] OR "phase III"[Title/Abstract] OR "phase IV"[Title/Abstract] OR "mass vaccination"[Title/Abstract])

Sanofi/GSK VAT00002 Search Strategy

"VAT00002"[Title/Abstract] AND ("COVID-19"[MeSH Terms] OR "COVID-19 Vaccines"[MeSH Terms] OR "SARS-CoV-2"[MeSH Terms]) AND ("clinical trials, phase iii as topic"[MeSH Terms] OR "clinical trials, phase iv as topic"[MeSH Terms] OR "Clinical Trials as Topic"[MeSH Terms] OR "phase 3"[Title/Abstract] OR "phase 4"[Title/Abstract] OR "efficacy"[Title/Abstract] OR "effectiveness"[Title/Abstract] OR "phase III"[Title/Abstract] OR "phase IV"[Title/Abstract] OR "mass vaccination"[Title/Abstract])

Moderna mRNA-1273

"mRNA-1273"[Title/Abstract] AND ("COVID-19"[MeSH Terms] OR "COVID-19 Vaccines"[MeSH Terms] OR "SARS-CoV-2"[MeSH Terms]) AND ("clinical trials, phase iii as topic"[MeSH Terms]

OR "clinical trials, phase iv as topic"[MeSH Terms] OR "Clinical Trials as Topic"[MeSH Terms] OR "phase 3"[Title/Abstract] OR "phase 4"[Title/Abstract] OR "efficacy"[Title/Abstract] OR "effectiveness"[Title/Abstract] OR "phase III"[Title/Abstract] OR "phase IV"[Title/Abstract] OR "mass vaccination"[Title/Abstract])

Additional safety monitoring search strategy

"covid 19 vaccines/adverse effects"[MeSH Terms] OR (("adverse effects"[Title/Abstract] OR "safety monitoring"[Title/Abstract] OR "safety profile"[Title/Abstract]) AND "covid 19 vaccine"[Title/Abstract])

2 Article selection flow diagram

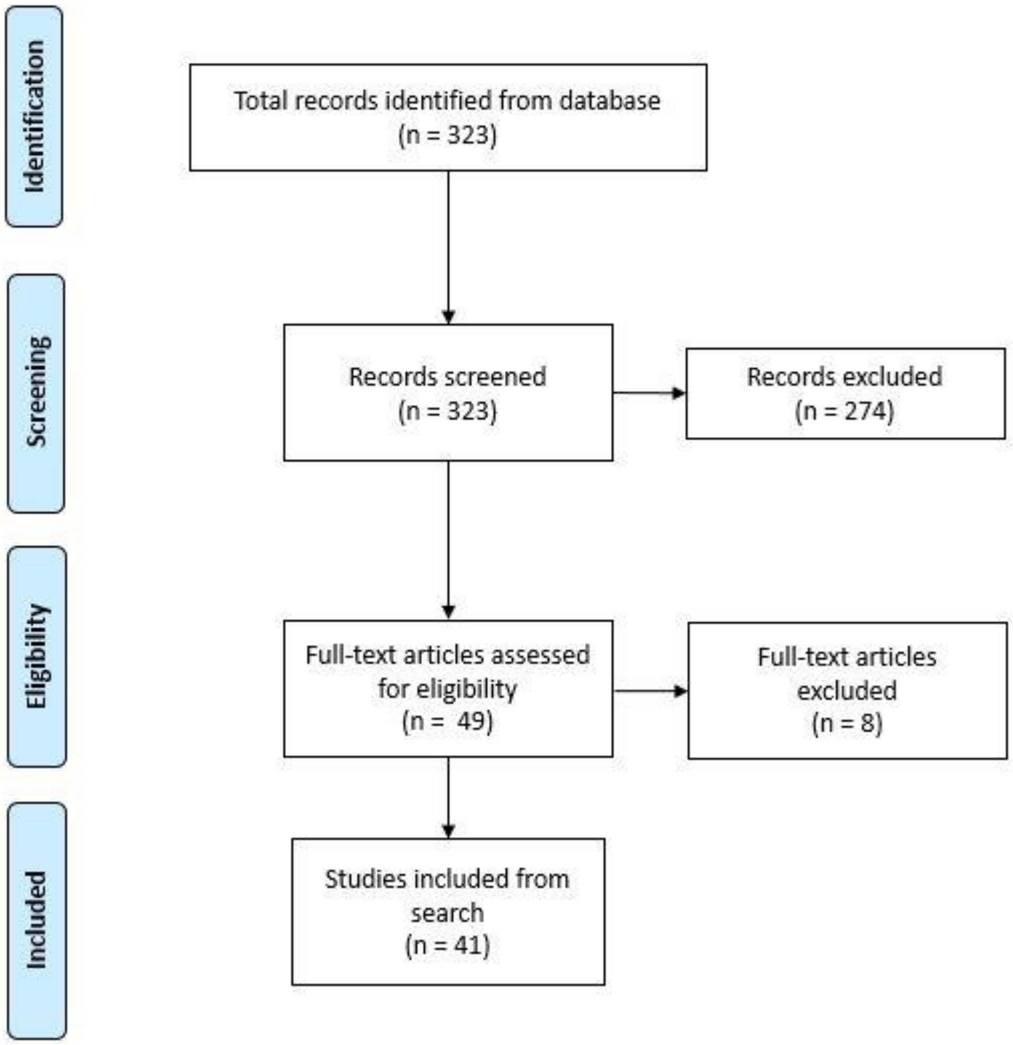

Appendix Figure 1: Article screening and selection flowchart
